# Role of dexmedetomidine in brain injury: a systematic review

**DOI:** 10.1007/s10787-025-01946-0

**Published:** 2025-09-30

**Authors:** Rania Zekry, Rehab H. Werida, Ehab S. Hegazy, Tarek M. Mostafa

**Affiliations:** 1 Department of Clinical Pharmacy & Pharmacy Practice, Faculty of Pharmacy, Damanhour University, Damanhour, 22514 Egypt; 2 Department of Critical Care Medicine, Damanhour Medical National Institute, GOTHI, Damanhour, Egypt; 3 Department of Clinical Pharmacy, Faculty of Pharmacy, Tanta University, Tanta, Egypt

**Keywords:** Dexmedetomidine, Brain injury, Neuroprotection Anti-inflammatory

## Abstract

**Background:**

Brain injuries are major health concern worldwide, that have debilitating effects on patients and affect patients’ quality of life; therefore, we are in urgent need of identifying medications that have neuroprotective properties and help to maintain the patients’ cognitive function. Dexmedetomidine is a sedative medication commonly used in ICU situations; recent studies show that it may have neuroprotective properties via different mechanisms.

**Aim:**

The aim of this article is to review available literature regarding the impact of using dexmedetomidine in various types of brain injury.

**Method:**

A systematic review of the published papers on the PubMed and google scholar that investigated the effects of dexmedetomidine on patients with different brain injuries was employed.

**Results:**

A total of 17 papers were included in this review, of which 1 was a case report, 6 were animal studies, 1 was a retrospective descriptive study, 2 were retrospective cohorts, 5 were randomized controlled trials, 1 was a scoping review, and 1 was a meta-analysis. These papers demonstrated the effects of dexmedetomidine on patients with traumatic brain injury, intracerebral hemorrhage, ischemic brain injury, status epilepticus, and on patients undergoing craniocerebral surgeries.

**Conclusion:**

Dexmedetomidine is a very promising drug to be used in different types of brain injuries and in craniocerebral surgeries because it has demonstrated impressive neuroprotective properties by a variety of mechanisms.

**Graphical Abstract:**

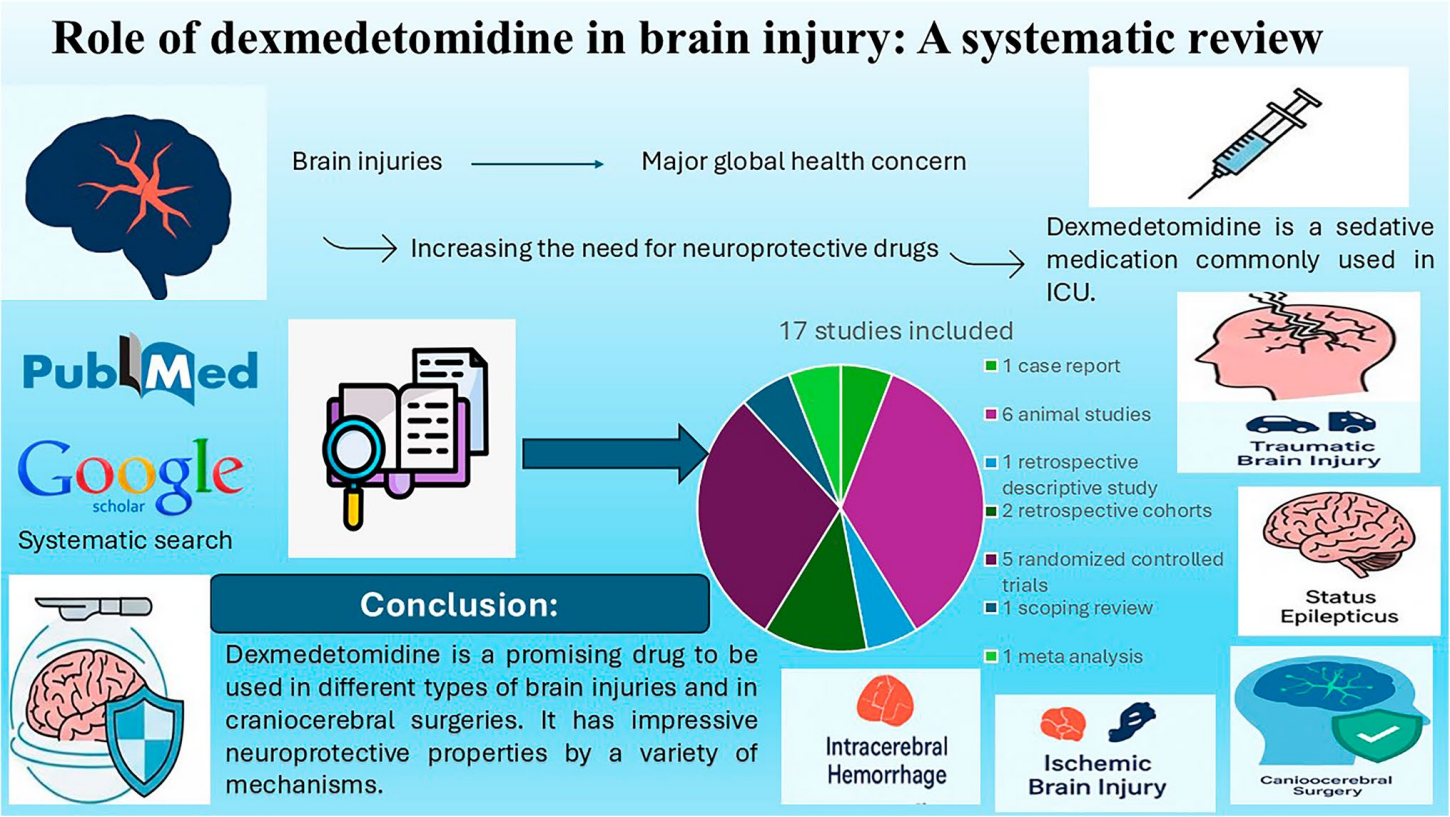

## Introduction

Brain injury is a public health concern all over the world, it may lead to disabilities or death, and impose a socioeconomic burden in every country. There are several types of brain injuries that are mainly classified into traumatic brain injury and non-traumatic brain injury. Non-traumatic brain injury can be further subdivided into ischemic stroke, hemorrhagic stroke, CNS infections like meningitis and encephalitis, aneurysmal subarachnoid hemorrhage, intracerebral hemorrhage, and cerebral ischemia and hypoxia (Headway, n.d.).

The consequences of brain injuries on patients’ life are variable depending on the location, the type, and the extent of injuries. Acute effects of brain injury on patients may include admission to intensive care unit (ICU), seizures, and infections. Long-term effects of brain injuries may influence patients’ physical activity, emotions, behaviors, cognitive functions, executive functions, and communication (Headway, n.d.).

Treatment of brain injury varies according to its cause; however, the main goal of treatment is to prevent further damage, allowing the brain to recover from the effects of the injury (Headway, n.d.). Neuroprotection is an important aspect of the management of brain injuries that should be emphasized to reduce acute and long-term consequences of brain injuries.

Dexmedetomidine is a non-opioid drug used to manage pain and sedation in ICU and during the peri-operative periods. The drug also has off-label uses which include therapy for insomnia, adjunctive analgesia, treatment and prevention of delirium (Reel and Maani 2023).

Dexmedetomidine is a highly selective alpha-2 agonist having a reversible sedative, anxiolytic, hypnotic, analgesic and sympatholytic properties (Reel and Maani 2025). Dexmedetomidine has the advantage of causing minimal respiratory depression (Hu et al. 2022).

Because TBI mortality is increased by systemic insults such as hypotension, hypoxia, and hypercarbia, it is imperative that those with TBI avoid subsequent secondary brain injury (Vella et al. 2017). Autonomic dysfunction is one of the feared but quite frequent extracranial consequences that can arise from moderate-to-severe TBI (Krishnamoorthy et al. 2021; Sykora et al. 2016); it is thought that the ensuing catecholamine surge causes various organ systems to fail downstream (Kôiv et al. 1997; Rosner et al. 1984).

In individuals with TBI, dexmedetomidine has shown promise as an alternate sedative. FDA-approved dexmedetomidine is a selective alpha-2 adrenergic receptor agonist that can be used to sedate non-intubated patients before or during surgery, as well as intubated and mechanically ventilated patients in intensive care units. Despite the potential risk of bradycardia and hypotension (Weerink et al. 2017), dexmedetomidine differs from conventional sedatives in a number of ways. It lowers blood pressure and heart rate (HR) without causing significant respiratory depression by decreasing sympathetic outflow from the central nervous system (Humble et al. 2016; Kamtikar and Nair 2019).

Recent study illustrated that when taken either alone or in combination with other medications, dexmedetomidine seems to have a hemodynamic safety profile that is comparable to that of conventional sedation regimens through reducing agitation episodes and may help to relieve sympathetic hyperactivity symptoms, despite the fact that it can cause brief bradycardia and hypotension episodes (Hatfield et al. 2024).

Recently, there are some studies establishing its safety and efficacy and explaining its effects on different types of brain injuries. The purpose of this scoping review is to give a detailed description of the effects of dexmedetomidine usage and its consequences for hospitalized patients with brain injury.

## Method

### Search strategy

Extensive literature search was performed for articles in PubMed and Google Scholar databases, from January 2000 till November 30, 2024. Keywords as “Dexmedetomidine”, “Brain injury”, “Intracranial hemorrhage”, “Traumatic” and “Status epilepticus” were used in varied ways, using “and” as search operator. Supplemental search was performed in cited references.

### Inclusion criteria

- Type of participants

This review considered studies that demonstrated the effects of dexmedetomidine on patients or rats with different types of brain injury such as traumatic brain injury, intracerebral hemorrhage, status epilepticus, and ischemic brain injury, it also included papers demonstrating dexmedetomidine effects on craniocerebral surgeries and on patients with traumatic brain injuries.

- Types of studies

This review considered any animal studies, case report, cross-sectional studies, observational studies, retrospective analysis, systematic review and meta-analysis demonstrating the effects of dexmedetomidine of different types of brain injury.

### Exclusion criteria

This review excluded papers that had not fitted the scope of the review, and those not published in English, or no English translation is available for them.

## Results

**Fig. 1:**
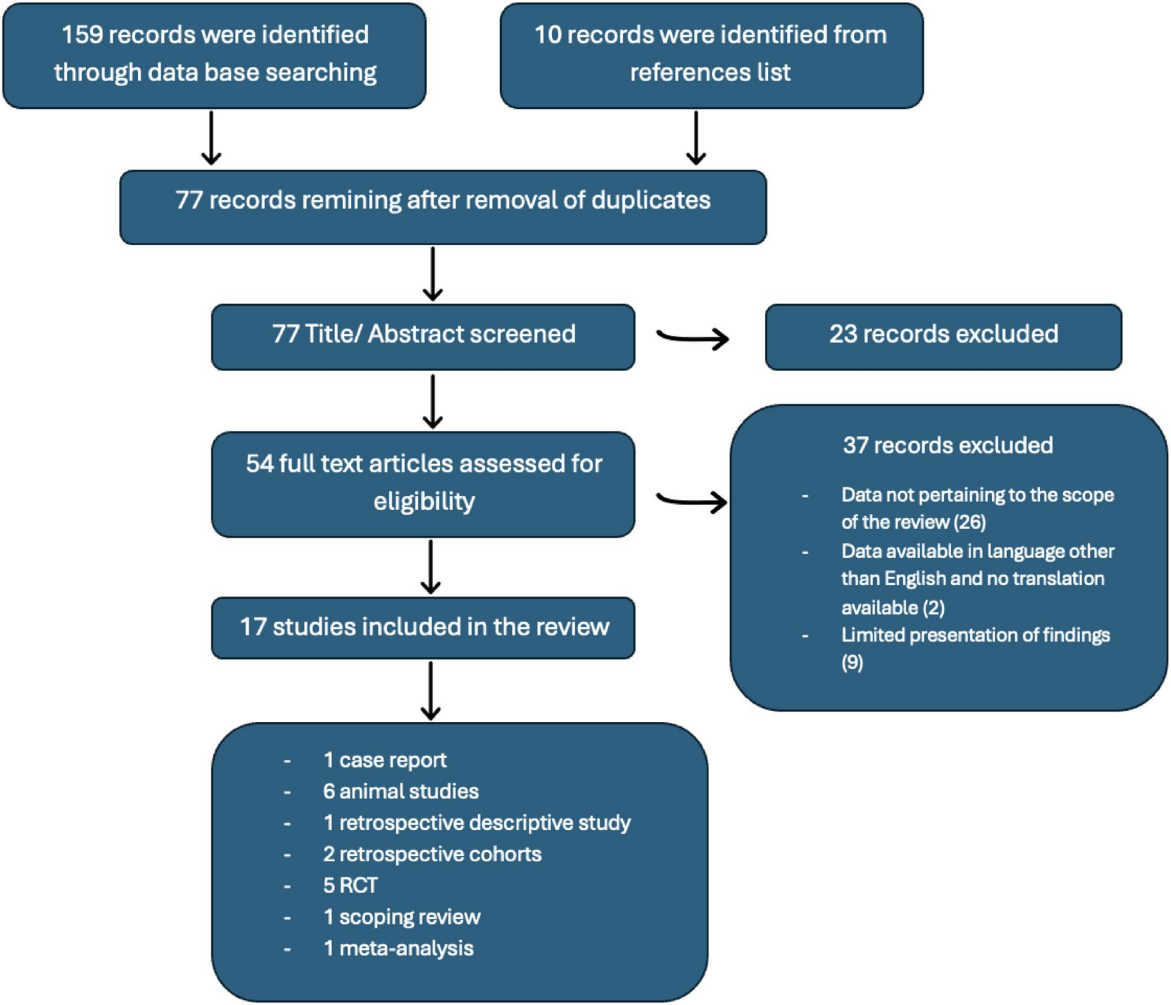
Flow chart of systematic review selection process

The search in PubMed yielded 70 results, and that in google scholar yielded 89 results. The search on references list yielded one result. After meeting inclusion and exclusion criteria and removal of duplicate, the search produced 17 results. The selection process of studies to be included in the systematic review is shown in Fig. 1.

Of the 17 results, 1 is case report, 6 are animal studies, 1 is a retrospective descriptive study, 2 are retrospective cohort, 5 are randomized controlled trials, 1 is a scoping review, and 1 is a meta-analysis as shown in Table 1.

**Table 1:** Summary of studies included in the systematic review

Reference, country	Design, follow-up, sample size	Outcome	Findings
*Case reports*			
Kubota et al., Japan (Kubota et al. 2013)	Case report.	Epileptic seizures.	Dexmedetomidine induced both epileptic seizures and non-epileptic movement in neonates during artificial ventilation.
*Animal studies*			
Jeon et al., Korea (Jeon et al. 2023)	120 rats divided into 3 groups (30 rats each) sham group, dexmedetomidine group, and propofol group, and followed for 14 days.	Brain-derived neurotrophic factor level in the cerebrospinal fluid (c-BDNF) and mechanical allodynia in a mild traumatic brain injury.	Dexmedetomidine intrathecal administration is associated with the reduction of TBI-induced mechanical allodynia through the immediate inhibition of (c-BDNF).
Hwang et al*.* Korea (Hwang et al. 2013)	40 rats followed for 14 days, divided into 5 groups (8 rats in each group, the groups are normal rats, sham operated rats, and the other 3 groups were ICH-induced injury group receiving different doses of dexmedetomidine 1, 5, or 10 μg/kg.	Short-term and spatial learning memory, and apoptosis.	Dexmedetomidine has significantly reduced ICH-induced impairment of short-term memory and spatial learning memory and expressed anti-apoptotic properties.
Eser et al. Turkey (Eser et al. 2008)	18 rats divided into 3 groups equally (6 rats in each group), sham group, the other 2 groups were prepared for transient global cerebral ischemia, and the second group received normal saline, and the third group received dexmedetomidine.	Neuroprotective effects on oxidant–antioxidant systems, pro-inflammatory cytokine tumor necrosis factor-α (TNF-α), and number of apoptotic neurons on hippocampus after transient global cerebral ischemia/reperfusion (I/R) injury.	Dexmedetomidine has significantly reduced the levels of tumor necrosis factor-α (TNF- α) and have neuroprotective effects in ischemia.
Kan et al., China (Kan et al. 2013)	65 rats followed for 14 days. The 65 rats were divided into 5 groups (group 1 = 15 rats, self-sustaining status epilepticus (SSSE) plus low-dose dexmedetomidine, group 2 = 15 rats SSSE plus high-dose dexmedetomidine, group 3 = 15 rats electrically stimulated non-dexmedetomidine injected group, group 4 = 10 rats electrode implanted but non-electrically stimulated rats, group 5 = 10 rats without any treatment.	Anticonvulsant effect in SSSE.	Dexmedetomidine has significantly reduced the number and the cumulative time of repeated seizures in rats with self-sustaining status epilepticus (SSSE).
Whittington et al., USA (Whittington et al. 2002)	16 rats received cocaine infusion to induce seizures, then divided into 2 groups (group 1 = 8 rats received dexmedetomidine, while group 2 = 8 rats received saline).	Altering the threshold for cocaine-induced seizure activity in rats.	Dexmedetomidine affectively increased seizures threshold in cocaine-induced seizures.
Xu et al., China (Xu et al. 2018)	followed for 5 days.60 rats divided into 2 groups (30 rats in each group dexmedetomidine group and control group), followed for 5 days.	Cognitive function and neuroinflammation in convulsive status epilepticus (CSE) rats	Dexmedetomidine significantly reduced seizures activity and increased long-term potentiation of convulsive status epilepticus (CSE).
*Retrospective descriptive study*			
Humble et al., USA (Humble et al. 2016)	Retrospective descriptive study included 85 adult patients with severe Traumatic brain injury (TBI) who received dexmedetomidine infusions in the Trauma Intensive Care Unit (ICU.	To describe the use of dexmedetomidine in adult ICU patients with severe TBI.	Dexmedetomidine reduced systolic and diastolic blood pressure, MAP, and heart rate, during infusion. And improved Richmond-agitation sedation scale (RASS) and Glasgow coma scale (GCS).
*Retrospective cohort*			
Khalili et al., Iran (Khalili et al. 2023)	Retrospective cohort included 138 patients divided into 2 groups. 69 patients in each group, one group received the routine sedation in ICU, while the other group received dexmedetomidine.	Hospital and ICU length of stay.	Dexmedetomidine is associated with significant reduction of ICU and hospital length of stay and significant improvement of patients’ functional outcomes
Xu et al., China. (Xu et al. 2022)	Retrospective cohort on patients with TBI, 2673 patients were included and divided into 2 group (175 were included in the dexmedetomidine group, and 2498 were included in the control group).	Effects on survival.	Dexmedetomidine significantly improved the survival of patients with traumatic brain injury.
*Randomized controlled trials*			
Yang et al. China (Yang et al. 2020)	Randomized controlled trial involved patients with acute brain injury, followed-up for 72 h sedation. 105 patients divided into 2 groups (56 patients were in dexmedetomidine group, while 49 patients received propofol).	Vital signs, acute physiology and chronic health evaluation II (APACHE II) score, Glasgow coma scale (GCS) score, bispectral index (BIS) value, artery blood gas analysis, and duration of mechanical ventilation.	Heart rate (HR), and MAP reduced significantly in dexmedetomidine group. Also, Glasgow coma scale (GCS), bispectral index (BIS), APACHE II score, and oxygenation index (PaO_2_/FiO_2_) were significantly improved in dexmedetomidine group.
Hou et al. China (Hou et al. n.d.)	Randomized controlled study involved 70 patients, with craniotomy surgery for TBI, divided into groups (35 patients in each group: dexmedetomidine group and placebo group).	Hemodynamic and pro-inflammatory responses.	Dexmedetomidine is associated with reduced hemodynamic responses to intubation and surgical stimulation.
Yu et al. China (Yu et al. 2023)	Randomized controlled trial involved patients who underwent emergency trauma surgery followed-up for 1 month. The study involved 310 patients divided into 2 groups (154 in normal saline group and 156 patients in the dexmedetomidine group).	Protective effect on post-traumatic stress disorder (PTSD) in patients with trauma, who were undergoing emergency surgery.	Dexmedetomidine administration preoperatively is associated with a significant reduction in PTSD.
Shaboob et al. Egypt (Shaboob and Mostafa 2023)	Randomized controlled trial involved 76 patients, with TBI undergoing surgeries, divided into 2 groups (38 in dexmedetomidine group and 38 in placebo group) and were followed-up for 4 weeks postoperatively.	Postoperative cognitive function.	Dexmedetomidine is associated with ameliorated cognitive function impairment that may be aggravated after the surgery.
Dong et al. China (Dong et al. 2023)	Randomized controlled trial involved 80 patients, undergoing craniocerebral trauma surgery, divided into 2 groups (40 in one group received 0.5 μg/kg dexmedetomidine and 40 in the other group that received 1 μg/kg dexmedetomidine).	Hemodynamic and cerebral protective effects.	In patients with traumatic brain injury, a dosage of dexmedetomidine at 0.5 μg/kg has a higher protective impact on brain function and is stable in hemodynamics.
*Scoping review*			
Hatfield et al. USA (Hatfield et al. 2024)	A scoping review included 11 articles, 2 of which are randomized controlled trials and 9 are observational studies.	The primary outcomes were the effects of dexmedetomidine on cerebral physiology, systemic hemodynamics, delirium, agitation, and paroxysmal sympathetic hyperactivity (PSH). It also studied adverse effects of dexmedetomidine.	The effects of dexmedetomidine on cerebral physiology are that dexmedetomidine did not show clinically significant differences in intracranial pressure (ICP) deviations. Dexmedetomidine was reported to cause hypotension and rare bradycardia. Dexmedetomidine is associated with lower agitation, delirium, and PSH.
*Meta-analysis*			
Jiang et al., China (Jiang et al. 2017)	Meta-analysis included 19 randomized controlled trials, included 879 patients.	Protective effects of dexmedetomidine on ischemic brain injury.	Dexmedetomidine is associated with maintaining intracranial homeostasis and reducing ischemic brain injury.

### Traumatic brain injury (TBI)

Humble et al*.* showed that dexmedetomidine administration is associated with a significant reduction in systolic and diastolic blood pressure, mean arterial pressure (MAP), and heart rate, during drug infusion. They have shown that once infusion has been stopped, systolic, diastolic, and mean arterial pressure and heart rate have returned to their pre-infusion level. They have also explained that this reduction was statistically significant but not clinically significant. Also, Richmond-agitation sedation scale (RASS) and Glasgow coma scale (GCS) have improved from pre-infusion to infusion time periods (Humble et al. 2016).

Yang et al. compared the effects of dexmedetomidine with standard therapy in patients with acute brain injury, and they found that heart rate (HR) and MAP reduced significantly in dexmedetomidine group compared to the standard therapy group. Also, Glasgow coma scale (GCS), bispectral index (BIS), APACHE II score, and oxygenation index (PaO_2_/FiO_2_) were significantly improved in dexmedetomidine group compared to the other control group. Dexmedetomidine group also showed significantly shorter duration of mechanical ventilation and no unexpected extubation, which has happened in the standard therapy groups. All these findings suggest that dexmedetomidine is safe, effective, and improve neurological functions in acute brain injury (Yang et al. 2020).

Khalili et al*.* showed that the administration of dexmedetomidine in patients with moderate-to-severe traumatic brain injury is associated with significant reduction of intensive care unit and hospital length of stay and significant improvement of patients’ functional outcomes as indicated by 6-month Glasgow outcome scale extended (GOSE) compared with placebo group (Khalili et al. 2023).

Xu et al. have found that dexmedetomidine significantly reduced in-hospital death among patients with traumatic brain injury. Dexmedetomidine significantly improved the survival of patients with traumatic brain injury irrespective of the starting time, the dose, or the duration of use (Xu et al. 2022).

Jeon et al. have found in a study that they have performed on rats that dexmedetomidine intrathecal administration is associated with the reduction of TBI-induced mechanical allodynia for up to 2 weeks after injury through the immediate inhibition of brain-derived neurotrophic factor level in the cerebrospinal fluid (c-BDNF), this inhibition also reduces the occurrence of TBI-induced chronic neuropathic pain (Jeon et al. 2023).

Hatfield et al*.* have found in their review that dexmedetomidine is a safe and an effective sedative in traumatic brain injury patients. They have explained multiple aspects of dexmedetomidine effects including its effect on cerebral physiology and how it is not clinically different from other sedative on Intracranial Pressure (ICP) deviations. They have demonstrated that dexmedetomidine has significantly reduced agitation and delirium, and that RASS was significantly improved and maintained within target levels when dexmedetomidine was used in traumatic brain injury patients. They have also explained the adverse effects of dexmedetomidine on systemic hemodynamics and showed that dexmedetomidine may cause reduction in systolic blood pressure, mean arterial pressure, and heart rate during its infusion (Hatfield et al. 2024).

### Intracerebral hemorrhage (ICH)

Hwang et al*.* have performed a study on rats in which they have induced intracranial hemorrhage (ICH) to study the effects of dexmedetomidine. They have found that dexmedetomidine has significantly reduced ICH-induced impairment of short-term memory and spatial learning memory by expressing anti-apoptotic properties through increasing brain-derived neurotrophic factor (BDNF) expression in the hippocampus of the rats. They suggested that dexmedetomidine may be used as a therapeutic agent for the conservation of memory function in patients with ICH (Hwang et al. 2013).

### Ischemic brain injury (IBI)

Eser et al. have performed a study in rats in which they have made a transient global ischemic/reperfusion injury and used dexmedetomidine to assess its neuroprotective effect. The results have shown that dexmedetomidine has significantly reduced the levels of tumor necrosis factor-α (TNF-α), malondialdehyde (MDA), and nitric oxide (NO), while increased the activity of superoxide dismutase (SOD), and catalase (CA), and significantly reduced the number of apoptotic neurons. These findings show the neuroprotective effects of dexmedetomidine in ischemia (Eser et al. 2008).

Jiang et al*.* have found that dexmedetomidine is associated with reduction in the release of neuroendocrine hormones and inflammatory mediators, it also maintains intracranial homeostasis, and reduces ischemic brain injury, which suggest its neuroprotective properties (Jiang et al. 2017).

### Status epilepticus

Whittington et al*.* have studied the effects of dexmedetomidine on cocaine-induced seizures in rats and have found that dexmedetomidine effectively increases seizures threshold in cocaine-induced seizures by reducing the response of extracellular dopaminergic neurotransmitters response to cocaine (Whittington et al. 2002).

Kan et al. have found that dexmedetomidine has significantly reduced the number and the cumulative time of repeated seizures in rats with self-sustaining status epilepticus probably through the reduction of the levels of Glutamate and glutathione/malondialdehyde (GSH/MDA) in hippocampus tissue (Kan et al. 2013).

Interesting findings were reported by Xu et al*.* study which state that dexmedetomidine significantly reduced the seizures activity, increased the long-term potentiation of convulsive status epilepticus, and improved the spatial cognitive function in rats with convulsive status epilepticus (Xu et al. 2018).

However, one case report published by Kubota et al*.* suggested that dexmedetomidine is inducing both epileptic seizures and non-epileptic movement in neonates during artificial ventilation (Kubota et al. 2013).

### Craniocerebral operations

Hou et al*.* found that the preoperative administration of dexmedetomidine is associated with reduced hemodynamic responses to intubation and surgical stimulation as MAP and heart rate were within the desirable range during the operation unlike the control group. They also found that dexmedetomidine significantly reduced the level of Tumor Necrosis Factor-alpha (TNF- α), Neuron-Specific Enolase (NSE), and Interleukin-6 (IL-6), which indicated that it reduces the inflammatory and immunologic reaction during the surgical operation (Hou et al. n.d.).

Another study performed by Yu et al*.* have demonstrated that dexmedetomidine administration preoperatively is associated with a significant reduction in the posttraumatic stress disorder (PTSD), compared with placebo group (Yu et al. 2023).

The finding of Shaboob et al*.* study demonstrated that the preoperative administration of dexmedetomidine is associated with a reduction in the inflammatory and oxidative response to surgery, with ameliorated cognitive function impairment that may be aggravated by the surgery (Shaboob and Mostafa 2023).

Dong et al*.* have compared different doses of dexmedetomidine and their effect on patients undergoing craniocerebral trauma surgery; and found that although both doses (0.5 μg/kg and 1 μg/kg) provided a protective effect on brain function compared to placebo group, the lower dose which was 0.5 μg/kg showed better hemodynamics and protective function (Dong et al. 2023).

## Discussion

Several studies have been performed and demonstrated that dexmedetomidine has beneficial neurological and hemodynamic effects on different types of brain injuries.

There are many animal studies that have performed to explain the mechanisms by which dexmedetomidine exerts its beneficial and neuroprotective effects.

Hu et al*.* summarized the mechanisms by which dexmedetomidine exerts its neuroprotective effects (Hu et al. 2022). These mechanisms are:

A. *Reducing inflammation:* dexmedetomidine reduces inflammation by regulating inflammatory response, reducing inflammatory mediators and inflammasomes. It also inhibits the reactivity of T cells, macrophages, neutrophils, and microglia. In addition, it reduces oxidative stress-mediated inflammations.
B. *Protecting brain barriers and reduce cerebral edema:* dexmedetomidine increases the expression of tight junction proteins to maintain the integrity of  blood-brain barrier (BBB), it also inhibits the destruction of BBB by inflammatory process.
C. *Reducing cells apoptosis:* dexmedetomidine inhibits apoptosis induced by mitochondrial injury, weakens endoplasmic reticulum stress, inhibits apoptosis induced by oxidative stress, and it also increases the expression of anti-apoptotic proteins.
D. *Protecting cellular structures:* dexmedetomidine promotes mitosis and neural restoration and increases the expression of neurotrophic factors. It also protects the integrity of the membrane and nuclear structure and decreases histone release.
E. *Reducing autophagy:* reduces the production of autophagy-related proteins and interferes with autophagy signaling pathways (Hu et al. 2022; Marehbian et al. 2017).

Several human studies have been performed to evaluate the effects of dexmedetomidine in patients with different types of brain injuries and some of them compared its effects with the standard sedatives.

Brain injuries including traumatic brain injury, intracerebral hemorrhage, ischemic brain injury, and status epileptics, also brain surgeries are situations in which dexmedetomidine is studied, and its effects are reported as follows:

### Traumatic brain injury

Traumatic brain injury (TBI) is a major problem worldwide, because of its complicated symptoms and unfavorable outcomes on patients’ health. The management of this condition focuses on effectively controlling mean arterial pressure (MAP), intracranial pressure (ICP), cerebral perfusion pressure (CPP), seizures control, and other measures (Luo et al. 2010; Marehbian et al. 2017).

The pathophysiological changes that happen in traumatic brain injury include inflammation, microglial activation, and neuronal death; these changes eventually affect the cognitive function of the patients (Luo et al. 2010). Dexmedetomidine have shown to reduce all these pathological changes and facilitate neuronal recovery (Hu et al. 2022).

Dexmedetomidine administration in traumatic brain injury has been found to reduce MAP, HR, systolic and diastolic blood pressure. it also improved Richmond-agitation sedation scale (RASS), Glasgow coma scale (GCS), bispectral index (BIS), APACHE II score, and oxygenation index (PaO_2_/FiO_2_) (Humble et al. 2016; Yang et al. 2020).

Dexmedetomidine was also found to reduce the duration of mechanical ventilation, the length of stay in intensive care unit, and hospital’s length of stay with a significant improvement in patients’ functional outcomes as indicated by Glasgow outcome scale extended (GOSE). It has also improved patients’ survival and reduced the occurrence of TBI-induced chronic neuropathic pain (Jeon et al. 2023; Khalili et al. 2023; Xu et al. 2022).

Dexmedetomidine was also found to reduce agitation and delirium, and maintain RASS score within target levels. Its sympatholytic properties reduce catecholamines release, making it an ideal treatment for autonomic dysregulation and paroxysmal sympathetic hyperactivity which might happen in patients with severe traumatic brain injury, this results in improved outcomes of patients with traumatic brain injury.

### Intracerebral hemorrhage

Intracerebral hemorrhage (ICH) may result in an irreversible damage to the central nervous system affecting its function. Short-term and learning memory problems are the most encountered consequences of intracerebral hemorrhage (MacLellan et al. 2009; Xiong et al. 2016).

Animal studies have found that dexmedetomidine has significantly reduced ICH-induced impairment of short-term memory and spatial learning memory by expressing an anti-apoptotic properties. They suggested that dexmedetomidine may be used as a therapeutic agent for the conservation of memory function in patients with ICH (Hwang et al. 2013).

Further studies to reach a definitive conclusion regarding dexmedetomidine effects on human with ICH are warranted.

### Ischemic brain injury

Cerebral ischemia can result from cardiac arrest, ischemic shock, or complicating heart surgery. Ischemia causes several negative effects including decreased motor control, seizures, cognitive impairment, and may even extend to coma or death. During brain ischemia, the production of reactive oxygen species is increased in neurons (Busl and Greer 2010; Endesfelder et al. 2017; Eser et al. 2008).

Dexmedetomidine was found to reduce the release of neuroendocrine hormones, and inflammatory mediators, such as Tumor Necrosis Factor-alpha (TNF-α), Malondialdehyde (MDA), and Nitric Oxide (NO). It also increases the activity of Superoxide Dismutase (SOD) and Catalase (CAT). Dexmedetomidine maintained intracranial homeostasis and reduced ischemic brain injury. These findings demonstrate the neuroprotective effects of dexmedetomidine in ischemia (Eser et al. 2008).

### Status epilepticus

Status epilepticus is a medical emergency that could precipitate a permanent neurological damage and negatively affect cognitive function (Walker 2018).

Dexmedetomidine was found to effectively increase seizures threshold, it also reduced the number and the cumulative time of repeated seizures in status epilepticus and reduced seizures activity and improved spatial cognitive function (Kan et al. 2013; Whittington et al. 2002; Xu et al. 2018).

However, one case report revealed that dexmedetomidine is inducing both epileptic seizures and non-epileptic movement in neonates during artificial ventilation (Kubota et al. 2013).

Since there is conflicting evidence about the effect of dexmedetomidine in status epilepticus, further studies should be performed to clarify its effects.

### Craniocerebral operations

Craniocerebral operation may cause post-traumatic distress syndrome (PTSD), postoperative cognitive impairment, ischemic brain injury, or neurological dysfunction due to neuroendocrine reaction or neuroinflammation (Dong et al. 2023).

Dexmedetomidine is associated with reduced hemodynamic responses to intubation and surgical stimulation as MAP and HR, it is also found to reduce the level of NSE and IL-6, which indicated that it reduces the immunologic reaction during the operation (Hou et al. n.d.).

Dexmedetomidine administration preoperatively is associated with significant reduction in PTSD and cognitive function impairment that may be aggravated by the surgery (Shaboob and Mostafa 2023; Yu et al. 2023).

Numerous studies have demonstrated that dexmedetomidine has neuroprotective properties, including the ability to lower oxidative stress and the inflammatory response, inhibit apoptosis, shield the blood–brain barrier (BBB), preserve the equilibrium of the coagulation–anticoagulant system, and avoid vasospasm (Liu et al. 2022; Wang et al. 2016). The neuroprotective benefits of dexmedetomidine in preventing inflammatory reactions, lowering the release of neuroendocrine hormones, and preserving intracranial homeostasis were validated by a meta-analysis involving 879 patients (Wang et al. 2016). According to a previous published review, dexmedetomidine’s neuroprotective effects mostly included reducing autophagy and apoptosis, inhibiting inflammatory responses, preserving the blood–brain barrier, and promoting stable cell architectures. As a result, dexmedetomidine can give patients with brain injuries a significant edge in their neurological rehabilitation.

Dexmedetomidine has demonstrated potent neuroprotective benefits, including preserving hemodynamic stability, lowering neuronal mortality, protecting the blood–brain barrier, and lowering postoperative agitation and cognitive impairment (Tao et al. 2024).

Curiously, a recent meta-analysis by Gatica et al. (2023) discovered that dexmedetomidine may significantly alter the production of IL-1β and IL-6 in the central nervous system and could also reduce oxidative stress and apoptosis in vitro (Gatica et al. 2023).

In summary, dexmedetomidine guarantees improved sedation and analgesia during traumatic brain injury surgery, lowers postoperative complications, suppresses immune cell migration and activation, controls autophagy, and prevents neuroinflammation and neuronal death. Through a variety of methods, dexmedetomidine, is considered a strong neuroprotective drug, that may enhance cognitive performance, reduce brain damage in pathological and surgical circumstances, and offer neuroprotection in complicated disorders like sepsis. These results provide credence to the use of dexmedetomidine in critical care and neurosurgical anesthesia, especially when neuroprotective measures are required.

## Limitations

The most important limitation of this review is that it includes many animal studies, but the study of dexmedetomidine effects on certain brain injury types has not been well established in humans yet. Another limitation is that many questions are still needed to be answered regarding the effects of dexmedetomidine on brain injury, and whether it is the ideal sedative that should be recommended first in patients with brain injury. Future research on the neuroprotective impact of dexmedetomidine on various injury types will be crucial.

## Conclusion

Dexmedetomidine can be a very promising drug to be used in different types of brain injuries and in craniocerebral surgeries because it has demonstrated impressive neuroprotective properties by a variety of mechanisms.

## Data Availability

All data supporting the findings of this study are available within the paper and its supplementary information.
